# Retinal thickness in healthy Australian Aboriginal and Torres Strait Islander children

**DOI:** 10.1371/journal.pone.0273863

**Published:** 2022-08-30

**Authors:** Rebecca A. Cox, Scott A. Read, Shelley Hopkins, David Alonso-Caneiro, Joanne M. Wood

**Affiliations:** School of Optometry and Vision Science, Centre for Vision and Eye Research, Queensland University of Technology, Brisbane, Queensland, Australia; Massachusetts Eye & Ear Infirmary, Harvard Medical School, UNITED STATES

## Abstract

**Background:**

Understanding normative retinal thickness characteristics is critical for diagnosis and monitoring of pathology, particularly in those predisposed to retinal disease. The macular retinal layer thickness of Australian Aboriginal and/or Torres Strait Islander children was examined using spectral-domain optical coherence tomography.

**Methods:**

High-resolution macular optical coherence tomography imaging was performed on 100 Aboriginal and/or Torres Strait Islander children and 150 non-Indigenous visually healthy children aged 4–18 years. The imaging protocol included a 6-line radial scan centred on the fovea. Images were segmented using semi-automated software to derive thickness of the total retina, inner and outer retina, and individual retinal layers across the macular region. Repeated measures ANOVAs examined variations in thickness associated with retinal region, age, gender and Indigenous status.

**Results:**

Retinal thickness showed significant topographical variations (p < 0.01), being thinnest in the foveal zone, and thickest in the parafovea. The retina of Aboriginal and/or Torres Strait Islander children was significantly thinner than non-Indigenous children in the foveal (p < 0.001), parafoveal (p = 0.002), and perifoveal zones (p = 0.01), with the greatest difference in the foveal zone (mean difference: 14.2 μm). Inner retinal thickness was also thinner in Aboriginal and/or Torres Strait Islander children compared to non-Indigenous children in the parafoveal zone (p < 0.001), and outer retinal thickness was thinner in the foveal (p < 0.001) and perifoveal zone (p < 0.001). Retinal thickness was also significantly greater in males than females (p < 0.001) and showed a statistically significant positive association with age (p = 0.01).

**Conclusion:**

There are significant differences in macular retinal thickness between Aboriginal and/or Torres Strait Islander children and non-Indigenous children, which has implications for interpreting optical coherence tomography data and may relate to risk of macula disease in this population.

## Introduction

The emergence of Fourier domain optical coherence tomography (OCT) imaging has enabled in-vivo visualisation of individual retinal layers and enables early identification of subtle but significant structural changes indicative of the onset of retinal disease. In children and adolescents, a comprehensive understanding of the normal retinal structure using OCT imaging, and knowledge of the ocular and systemic factors which influence retinal thickness, is essential for identifying normal retinal development, and enables earlier detection of paediatric ocular diseases including diabetic retinopathy [1, 2]. For example, in adolescents with both type 1 and type 2 diabetes, neurodegenerative macular retinal thinning detected from OCT imaging has been shown to precede the more obvious clinical signs of diabetic retinopathy [1, 2].

When interpreting pathological changes in retinal thickness, it is important to consider confounding factors that can influence retinal thickness, such as axial length and ethnicity [3–7]. For example, in the Sydney Myopia Study, the macula of 6- and 12-year-old Caucasian children was found to be significantly thicker than that of East Asian children in both the foveal and parafoveal macular zones, measured using time-domain OCT imaging [4, 5]. Similarly, in the Correction of Myopia Evaluation Trial, Caucasian children were found to have a thicker macula compared to African American, Asian, and Hispanic children [6]. These findings highlight the importance of ethnicity specific retinal thickness databases to enable more accurate interpretation of OCT findings in different populations.

Despite the important role of OCT in detecting abnormalities in retinal structure, and the widespread use of OCT imaging in clinical practice, there is no retinal thickness data available for either Australian Aboriginal and/or Torres Strait Islander children or adults. It is critical to increase our understanding of normal retinal structure in Aboriginal and/or Torres Strait Islander Peoples given that Aboriginal and/or Torres Strait Islander children and adolescents have been shown to be at a significantly higher risk for developing type 2 diabetes compared to their non-Indigenous counterparts [8–10]. This study aimed to determine the baseline retinal thickness characteristics of healthy Aboriginal and/or Torres Strait Islander children and adolescents to improve the interpretation of retinal OCT findings in this population.

## Materials and methods

All students enrolled at one primary and secondary school in a rural town in South-East Queensland, Australia, were invited to participate in the study. These schools were selected based on the high proportion of Aboriginal and/or Torres Strait Islander students enrolled in each school. The study was conducted in accordance with the Tenets of the Declaration of Helsinki and was approved by the Queensland University of Technology Human Research Ethics Committee and the Queensland Government Department of Education. The local Community-Controlled Health Service and school community assisted in the development of the project and the School Principals provided written informed consent for the school communities to be approached to participate in the project. Written informed consent was obtained from a parent or guardian of each child prior to their participation. A questionnaire was completed by a parent or guardian to determine whether each child identified as Aboriginal and/or Torres Strait Islander, as well as whether the child had any medical conditions or previous medical or surgical treatment to their eyes.

Each child underwent a series of tests including measurement of best corrected visual acuity, accommodation and vergence function, ocular biometry measures using the Lenstar LS 900 optical biometer (Haag Streit AG, Koeniz, Switzerland), and OCT imaging of the macular retina. Cycloplegic autorefraction was then performed 30 minutes following instillation of one drop of 1% tropicamide in each eye. All measurements were conducted between 9am and 3pm.

The Heidelberg Spectralis (Heidelberg Engineering, Heidelberg, Germany) spectral-domain OCT (SD-OCT) was used to image the right macula of each child. This instrument uses a super-luminescent diode with a central wavelength of 870 nm, capturing 40,000 A-scans per second, to generate images with a digital axial resolution of 3.9 μm, and incorporates frame averaging which has been shown to improve the visibility and assessment of retinal layer boundaries [11]. With the participant’s left eye occluded, two macula radial scans were captured, each consisting of a series of six radial line scans, centred on the fovea, separated by 30°, with a line length of 20°. Each scan was taken in high-resolution mode and averaged 30 B-scans per line. The instrument’s enhanced depth imaging (EDI) mode was used which allows for improved visibility of the posterior retinal structures without impacting significantly on retinal thickness measures [12, 13]. Any scan that exhibited poor quality (quality index < 20 dB) or non-foveal fixation was excluded from the analysis. Of the 280 children who participated in the study, data for 27 children with systemic disease, ocular disease, strabismus, reduced visual acuity (> 0.2 logMAR), or a history of ocular surgery or injury were excluded, and data for three children were excluded due to poor image quality, leaving images from 250 children included in the analysis.

### OCT image analysis

**Fig 1:**
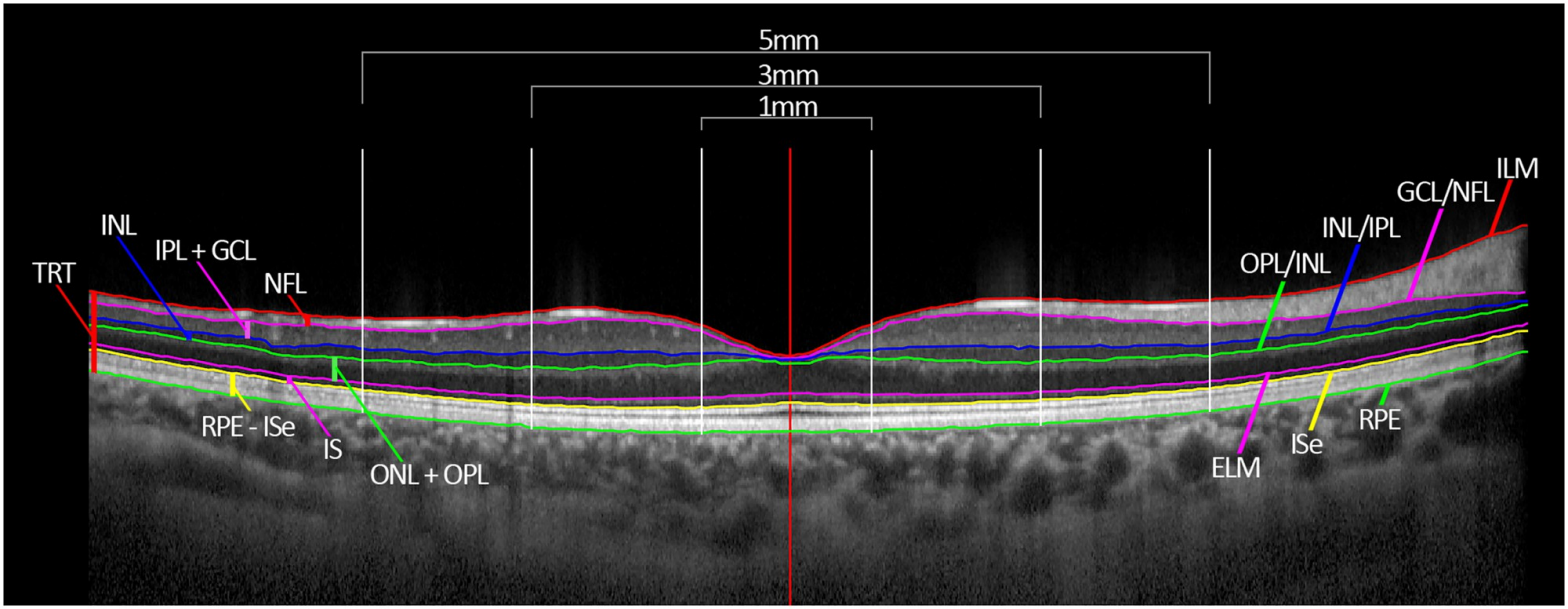
Example of the segmentation applied to each macula OCT image including the retinal layer boundaries: The internal limiting membrane (ILM), the ganglion cell layer (GCL)/nerve fibre layer (NFL) boundary, the inner nuclear layer (INL)/inner plexiform layer (IPL) boundary, the outer plexiform (OPL)/inner nuclear layer (INL) boundary, the external limiting membrane (ELM), the inner boundary of the inner segment ellipsoid band (ISe), and the outer boundary of the retinal pigment epithelium (RPE). Also shown are the total retinal thickness (TRT) measurement, and the individual layer thickness measurements including the inner retinal layers (the NFL, the IPL + GCL, and the INL), and the outer retinal layers (the outer nuclear layer (ONL) + OPL, the inner segment (IS), and the RPE to ISe layer).

Each OCT image was analysed using semi-automated software [14] as shown in Fig 1. This allowed for segmentation of the following layer boundaries: the inner boundary of the internal limiting membrane (ILM), the boundary between the nerve fibre layer (NFL) and the ganglion cell layer (GCL), the boundary between the inner plexiform layer (IPL) and the inner nuclear layer (INL), the boundary between the INL and the outer plexiform layer (OPL), the inner boundary of the external limiting membrane (ELM), the inner boundary of the inner segment ellipsoid band (ISe), and the outer boundary of the retinal pigment epithelium (RPE). An experienced, masked observer checked the accuracy of the segmentation and made manual corrections of any segmentation errors detected. The transverse magnification of each scan was corrected to account for ocular magnification using the individual ocular biometry and cycloplegic autorefraction data collected for each child, using previously described methods [15].

This segmentation process also allowed for measurements of individual layer thickness including: NFL thickness (from the ILM to the NFL/GCL boundary), IPL + GCL thickness (from the NFL/GCL boundary to the INL/IPL boundary), INL thickness (from the INL/IPL boundary to the OPL/INL boundary), ONL + OPL thickness (from the OPL/INL boundary to the ELM), inner segment (IS) layer thickness (from the ELM to the inner boundary of the ISe), and thickness of the RPE to ISe layer (from the inner boundary of the ISe to the outer boundary of the RPE). The total retinal thickness (TRT) was measured from the inner boundary of the ILM to the outer boundary of the RPE and the individual retinal layers were grouped into the inner retinal layers (including the NFL, IPL + GCL, and INL), and the outer retinal layers (including the ONL + OPL, IS, and RPE to ISe). Each thickness measurement was averaged across the foveal (central 1 mm zone), parafoveal (1 mm to 3 mm zone), and perifoveal zone (3 mm to 5 mm zone).

### Statistical analysis

All statistical tests were carried out using SPSS version 26.0 (SPSS Inc. IBM, Chicago, IL). Differences between Aboriginal and/or Torres Strait Islander children and non-Indigenous children in terms of demographic and ocular biometry were explored using independent samples T-tests (for continuous variables) or χ2 test (for categorical variables). For each of the retinal thickness metrics, a multivariate repeated measures analysis of variance (ANOVA) was used to examine differences in retinal thickness between Aboriginal and/or Torres Strait Islander children and non-Indigenous children including within-subject factors of retinal zone and retinal meridian, between-subject factors of Indigenous status and gender, with age and axial length included as covariates. Parameter estimates describing the relationship (β values and their significance) between the thickness measures and the covariates were also calculated within the ANOVA. Bonferroni adjusted pairwise comparisons examined the significant main effects and interactions. Additional repeated measures ANOVAs were conducted including spherical equivalent refractive error (SER) as a covariate instead of axial length. All results are presented as the mean ± standard deviation.

## Results

Two hundred and fifty participants were included in the analysis. Mean age was 11.7 ± 3.4 years (range: 4.9 to 18.2 years), with 126 children (50%) being female. The mean SER was +0.72 D ± 0.70 D (range: -1.00 to +6.57 D), with four children having myopia of 0.50 D or more and six with hyperopia of 2.00 D or more. The mean axial length was 23.13 ± 0.73 mm (range: 20.59 to 25.26 mm). One hundred of the children (40%) identified as Aboriginal and/or Torres Strait Islanders. The demographic information and ocular biometric measures of the Aboriginal and/or Torres Strait Islander children and non-Indigenous children are presented in Table 1. There was no difference in the mean age (p = 0.08), axial length (p = 0.92), or gender balance (p = 0.88) of Aboriginal and/or Torres Strait Islander children and non-Indigenous children, however the mean SER of Aboriginal and/or Torres Strait Islander children was significantly less hyperopic compared to non-Indigenous children (Aboriginal and/or Torres Strait Islander: +0.52 ± 0.80 D; non-Indigenous: +0.86 ± 0.58 D: p < 0.001). The mean Quality Index score of the included OCT images was 30.9 ± 3.1 dB (Aboriginal and/or Torres Strait Islander: 30.5 ± 3.5 dB; non-Indigenous: 31.2 ± 2.9 dB: p = 0.113).

**Table 1:** Demographic information and ocular biometric measures of the Aboriginal and/or Torres Strait Islander children and non-Indigenous children.

	Mean ± SD*	
	Aboriginal and/or Torres Strait Islander (n = 100)	Non-Indigenous (n = 150)
Age (years)	11.21 ± 3.39	11.99 ± 3.41
Gender (% female)	51%	50%
Spherical Equivalent Refraction (D)	+0.52 ± 0.80	+0.86 ± 0.58
Axial length (mm)	23.12 ± 0.73	23.13 ± 0.73

*Standard deviation.

### Topographical distribution of retinal thickness

**Fig 2:**
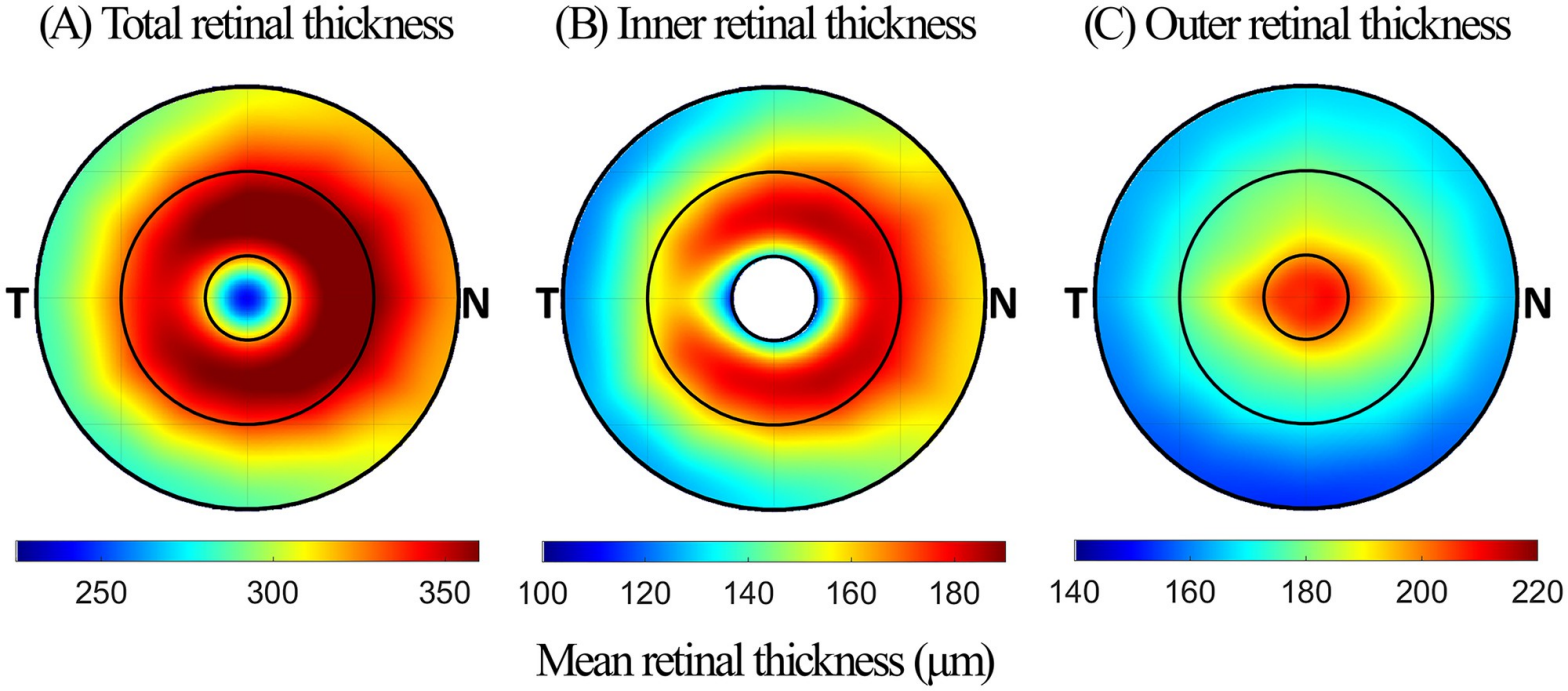
Thickness maps illustrating the average thickness of (A) the total retina, (B) the inner retinal layers, and (C) the outer retinal layers, across a 5 mm zone.

Fig 2 illustrates the topographical distribution of thickness across the 5mm macular zone for TRT (A), inner retinal thickness (B), and outer retinal thickness (C), for all participants. The mean values for TRT for all participants are presented in Table 2. TRT was thinnest in the foveal zone, followed by the perifoveal and parafoveal zones (p < 0.001 for all comparisons). Across all zones, the nasal retina was thickest, followed by the superior and inferior retina, while the temporal retina was the thinnest (p < 0.001 for all comparisons).

**Table 2:** Mean total retinal thickness (TRT) in each retinal zone and meridian, for all participants.

Retinal location	Total retinal thickness (μm) (n = 250)			
		Percentile		
	Mean ± SD	5th	95th	p-value
Foveal zone	266.5 ± 18.3	238.1	296.3	< 0.001
Parafoveal zone	351.4 ± 13.9	329.5	375.8	
Perifoveal zone	318.8 ± 12.8	296.4	338.2	
Superior retina	316.8 ± 12.8	297.4	338.9	< 0.001
Inferior retina	307.7 ± 13.7	284.6	330.7	
Nasal retina	320.8 ± 13.9	299.8	346.8	
Temporal retina	300.7 ± 13.0	280.6	323.6	

SD = Standard deviation.

**Fig 3:**
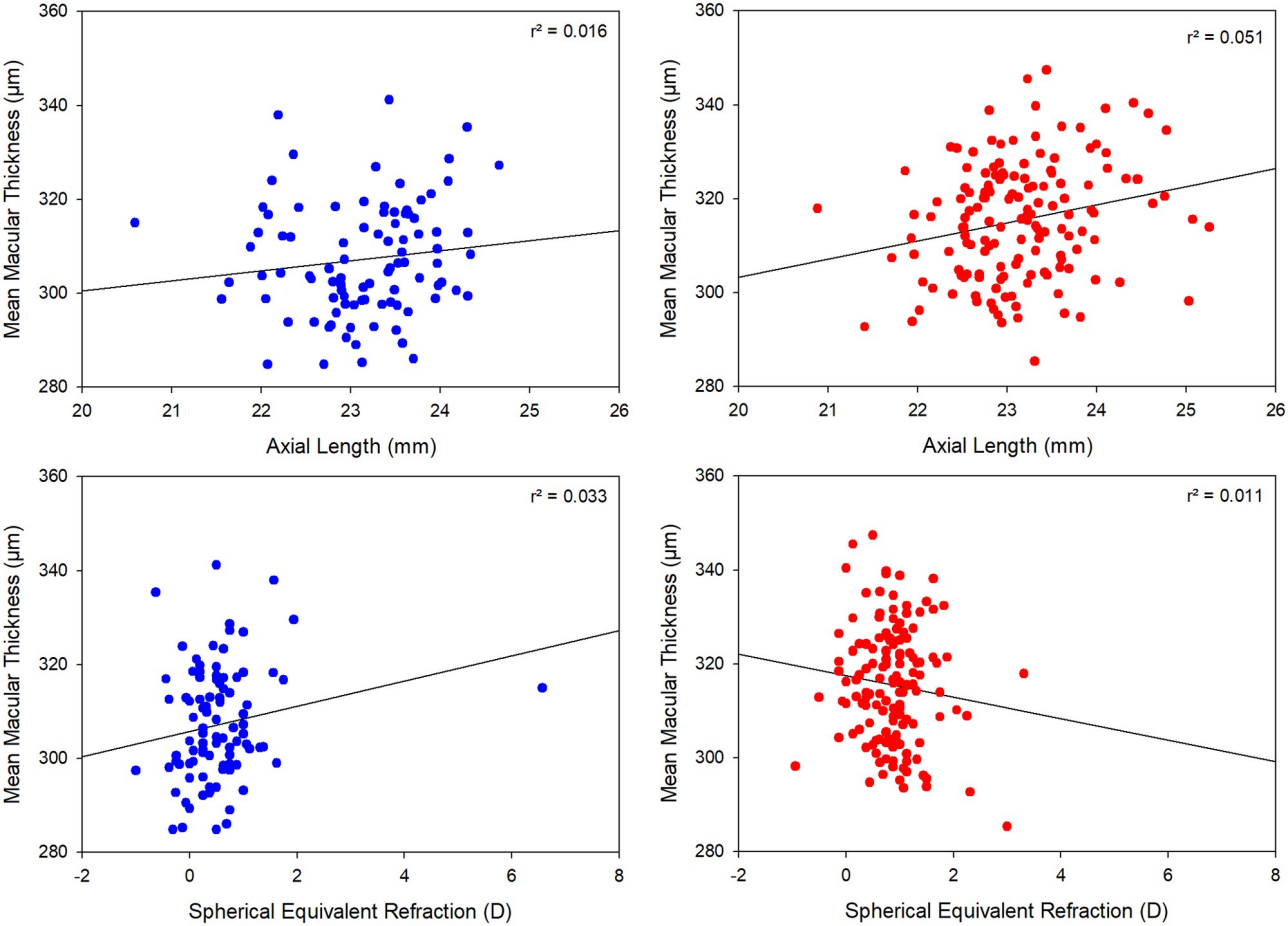
The relationship between the average macular retinal thickness and axial length (top) and between the average macular retinal thickness and spherical equivalent refraction (bottom) for Aboriginal and/or Torres Strait Islander children (left, blue symbols) and non-Indigenous children (right, red symbols).

The repeated measures ANOVAs revealed no significant effect of either axial length or SER on TRT, inner retinal thickness, or outer retinal thickness in any zone, and no significant interaction with Indigenous status (all p > 0.05). Fig 3 shows scatterplots of axial length and thickness and SER and thickness in this population. TRT was significantly thicker in males compared to females in the foveal (mean difference: 8.3 μm, p = 0.001), parafoveal (mean difference: 8.5 μm, p < 0.001) and perifoveal zones (mean difference: 5.8 μm, p = 0.001). The inner retinal layer thickness was also greater in males compared to females in the parafoveal (mean difference: 5.7 μm, p < 0.001) and perifoveal zones (mean difference: 3.2 μm, p = 0.01). The outer retinal layer thickness was not significantly different between genders in the foveal zone (mean difference: 2.0 μm, p = 0.1) but was significantly thicker in males in the parafoveal (mean difference: 2.6 μm, p = 0.02) and perifoveal zones (mean difference: 2.7 μm, p = 0.01).

There was a significant positive association between TRT and age in the foveal zone (β = 0.8 μm/year, p = 0.01) and in the parafoveal zone (β = 0.7 μm/year, p = 0.01), but not in the perifoveal zone (β = 0.2 μm/year, p = 0.41). Total inner retinal layer thickness was positively associated with age in the parafoveal zone (β = 0.5 μm/year, p = 0.01), while outer retinal layer thickness increased with age only in the foveal zone (β = 0.5 μm/year, p = 0.01). There was no significant interaction between Indigenous status and age for TRT (p = 0.84), outer retinal thickness (p = 0.55), or inner retinal thickness (p = 0.38), and this was the case for all retinal zones.

### Retinal thickness and Indigenous status

**Fig 4:**
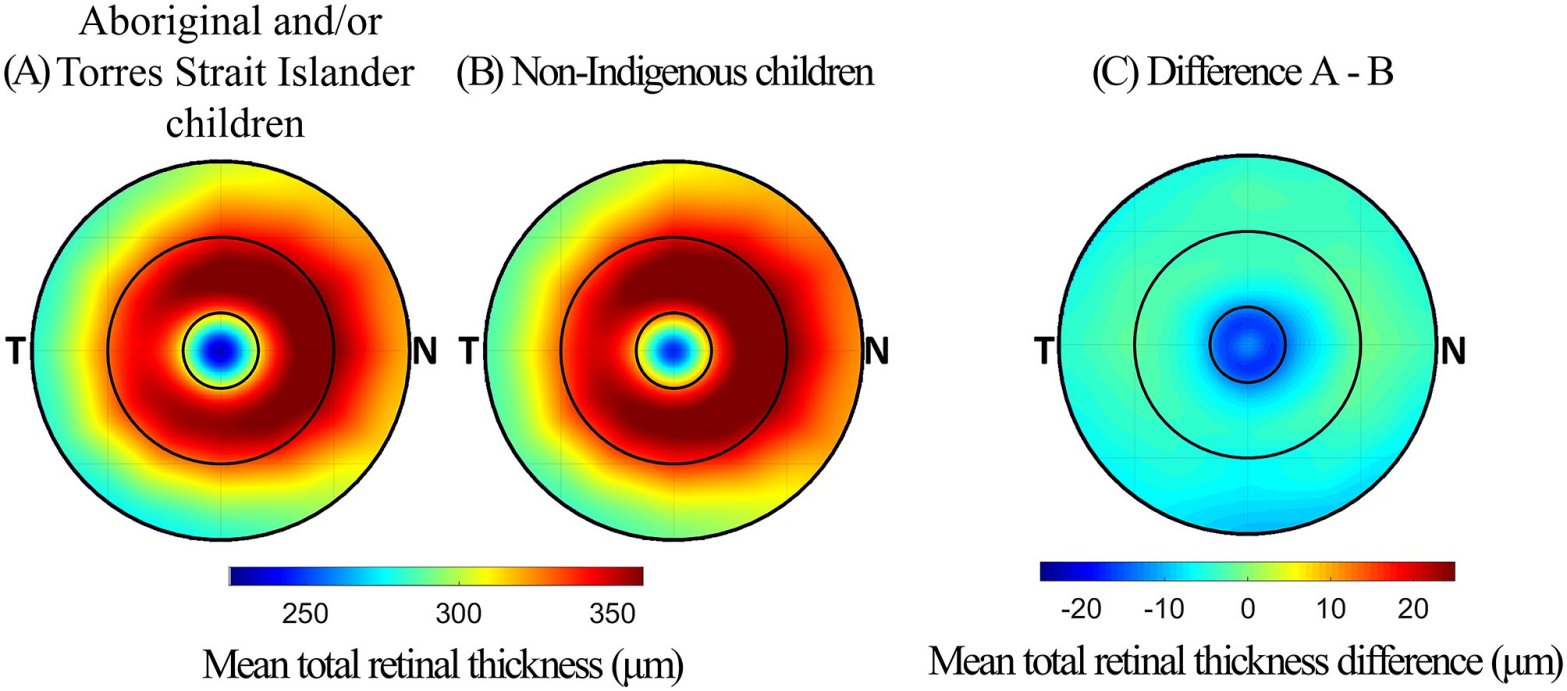
Thickness maps illustrating the mean TRT for (A) Aboriginal and/or Torres Strait Islander children, (B) non-Indigenous children, and (C) the difference between the two groups. Cooler colours (negative values) in the thickness difference map represents a thinner retina among Aboriginal and/or Torres Strait Islander children.

The mean TRT for Aboriginal and/or Torres Strait Islander children and non-Indigenous children in each retinal zone are presented in Table 3. The TRT of Aboriginal and/or Torres Strait Islander children was significantly thinner across the entire 5 mm zone and in each retinal zone, compared to non-Indigenous children (all p ≤ 0.01), with the difference being greatest in the foveal zone where the mean difference was 14.2 μm (p < 0.001). The difference in TRT between the two groups is illustrated in Fig 4.

**Table 3:** Mean total retinal thickness (TRT) across the entire 5 mm zone, and in each retinal zone for Aboriginal and/or Torres Strait Islander children and non-Indigenous children, and results of pairwise comparisons.

Retinal location	Total retinal thickness (μm)						
	Aboriginal and/or Torres Strait Islander (n = 100)			Non-Indigenous (n = 150)			
		Percentile			Percentile		
	Mean ± SD	5th	95th	Mean ± SD	5th	95th	p-value
All zones	307.2 ± 12.1	288.3	328.9	315.5 ± 12.3	295.4	336.7	< 0.001
Foveal zone	257.8 ± 16.4	235.2	286.0	272.0 ± 17.4	243.9	305.2	< 0.001
Parafoveal zone	347.7 ± 13.0	326.4	373.5	353.8 ± 14.0	331.8	377.3	0.002
Perifoveal zone	316.0 ± 13.1	293.5	343.0	320.6 ± 12.4	297.4	338.2	0.01

SD = Standard deviation.

The mean thickness for each individual retinal layer for Aboriginal and/or Torres Strait Islander children and non-Indigenous children are presented in Table 4. Aboriginal and/or Torres Strait Islander children had a significantly thinner inner retina compared with non-Indigenous children in the parafoveal zone (p < 0.001), and this was due to significant differences in the IPL + GCL (p < 0.001) and INL layers (p = 0.01), while there was no difference in NFL thickness between groups.

**Table 4:** Retinal thickness measurements of each retinal layer within the inner and outer retina, by retinal zone, and comparisons by Indigenous status.

			Aboriginal and/or Torres Strait Islander			Non-Indigenous			
				Percentile			Percentile		
	Retinal zone	Retinal layer	Mean ± SD	5th	95th	Mean ± SD	5th	95th	p-value
Inner retina	All zones	All layers	156.1 ± 8.4	142.9	169.3	158.9 ± 7.9	145.7	172.8	0.01
	Parafoveal zone	All layers	161.9 ± 9.0	147.6	177.0	166.6 ± 8.6	153.5	181.0	< 0.001
		NFL	29.4 ± 2.1	25.5	33.5	29.7 ± 2.1	26.4	33.2	0.58
		IPL + GCL	92.6 ± 5.9	84.0	102.2	96.1 ± 5.7	85.9	105.3	<0.001
		INL	39.8 ± 2.7	34.7	44.1	40.8 ± 2.7	36.7	45.5	0.01
	Perifoveal zone	All layers	150.3 ± 9.4	135.5	168.3	151.3 ± 8.5	137.2	166.0	0.47
		NFL	43.3 ± 3.7	37.5	49.8	43.5 ± 3.5	38.6	50.3	0.47
		IPL + GCL	69.9 ± 5.9	59.8	79.9	71.6 ± 5.5	62.7	80.4	0.06
		INL	34.0 ± 2.5	29.8	38.4	33.9 ± 2.3	30.5	38.3	0.50
Outer retina	All zones	All layers	185.1 ± 6.5	174.9	195.8	188.4 ± 7.6	176.4	201.1	0.001
	Foveal zone	All layers	203.0 ± 8.1	190.3	218.3	208.0 ± 9.4	192.7	222.7	< 0.001
		ONL + OPL	97.7 ± 7.6	85.1	111.2	102.7 ± 8.2	89.6	117.7	<0.001
		IS	29.3 ± 1.1	27.6	31.4	29.4 ± 1.2	27.2	31.1	0.71
		RPE to ISe	76.0 ± 2.6	71.8	80.5	75.9 ± 2.3	71.6	79.3	0.59
	Parafoveal zone	All layers	186.2 ± 6.9	175.2	198.9	187.6 ± 8.2	175.4	201.2	0.23
		ONL + OPL	93.4 ± 5.8	84.4	103.4	94.6 ± 6.6	84.5	106.2	0.14
		IS	24.0 ± 1.0	22.5	25.9	24.2 ± 1.0	22.6	25.8	0.06
		RPE to ISe	68.9 ± 2.2	65.6	72.8	68.7 ± 2.1	65.6	72.5	0.28
	Perifoveal zone	All layers	166.0 ± 6.8	153.6	179.4	169.5 ± 7.1	157.2	180.5	<0.001
		ONL + OPL	77.7 ± 5.8	68.2	89.0	81.0 ± 5.9	71.7	91.9	<0.001
		IS	21.0 ± 0.7	20.0	22.3	21.4 ± 0.6	20.2	22.6	<0.001
		RPE to ISe	65.2 ± 2.0	62.2	68.5	65.6 ± 2.0	62.3	69.0	0.17

SD = Standard deviation.

**Fig 5:**
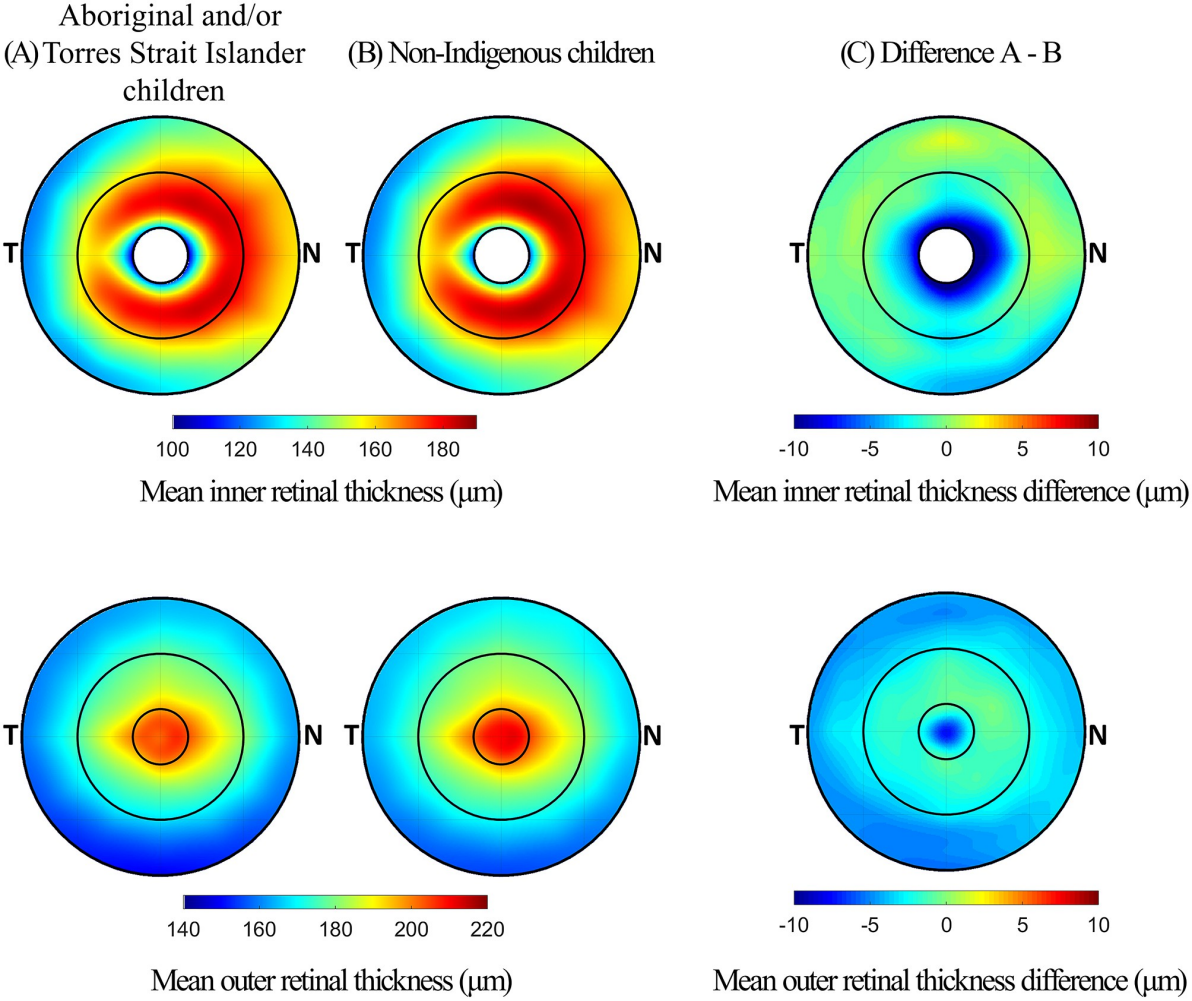
Mean inner retinal layer thickness (top) and outer retinal layer thickness (bottom) for (A) Aboriginal and/or Torres Strait Islander children and (B) non-Indigenous children, and (C) the differences between the two groups.

The outer retinal layers of Aboriginal and/or Torres Strait Islander children were significantly thinner than those of non-Indigenous children in both the foveal (p < 0.001) and perifoveal zones (p < 0.001). The difference in foveal zone thickness was primarily due to a difference in ONL + OPL layer thickness (p < 0.001), and the difference in perifoveal zone thickness was due to the ONL + OPL layer (p < 0.001) and to a smaller extent IS layer thickness differences (p < 0.001). There was no difference in RPE to ISe thickness between the two groups. The differences in inner and outer retinal layer thickness between Aboriginal and/or Torres Strait Islander children and non-Indigenous children are illustrated in Fig 5.

## Discussion

This research presents the first analysis of retinal thickness characteristics of Australian Aboriginal and/or Torres Strait Islander children and provides the first step in establishing normative characteristics of macular retinal thickness in this population. The findings demonstrate that Aboriginal and/or Torres Strait Islander children have a thinner macular retina compared to non-Indigenous children, with the most prominent differences evident in the foveal zone. Given the widespread use of OCT in both clinical and research settings, understanding these differences in normal retinal characteristics will facilitate earlier and more accurate detection of retinal thickness abnormalities in Aboriginal and/or Torres Strait Islander children by providing ethnicity specific, normative data.

The distribution of macula thickness across the central 5 mm macula reported in the current study is consistent with previous research using SD-OCT in primarily Caucasian Australian children, being thinnest in the foveal zone, followed by the perifoveal zone, and thickest in the parafoveal zone [16]. Meridional variations across all zones also followed the pattern previously described in visually normal children, with the nasal retina being thickest, and temporal retina being the thinnest [4, 16, 17]. Each individual retinal layer also showed topographical variations in thickness consistent with previous paediatric studies [16, 18, 19].

The mean retinal thickness values reported here for both Aboriginal and/or Torres Strait Islander children and non-Indigenous children are thicker than those reported in the Sydney Myopia Study, which used TD-OCT to image the retina [4, 17]. This is not unexpected given that TD-OCT has been shown to underestimate retinal thickness measurements by 65 to 70 μm compared to the Spectralis SD-OCT [20]. More recently, Read, Alonso-Caneiro (2017) reported similar values of retinal thickness to those found in the current study using the Heidelberg Spectralis SD-OCT [19]. In their cohort of 10- to 15-year-old children, the mean global retinal thickness of myopic children (mean SER -2.39 D) and non-myopic children (mean SER +0.35 D) were 308.8 μm and 312.4 μm, respectively. Interestingly, these values align closely with the mean values for Aboriginal and/or Torres Strait Islander children (307.2 μm) and non-Indigenous children (315.5 μm) in the current study. However, the between group differences in thickness in the current study are unlikely to be due to differences in refractive error, since the difference in SER between groups was only 0.34 D, compared to a difference of more than 2.50 D in the previous study [19]. Indeed, in the current study, we found no significant association between SER and retinal thickness, suggesting that differences in refractive error were not a confounder.

While previous studies have reported differences in TRT between different ethnic groups [3, 5, 6], this is the first study to report on the retinal thickness of Aboriginal and/or Torres Strait Islander children, who exhibited a significantly thinner retina than non-Indigenous children. It is not clear why differences in macula thickness exist between ethnic groups. Factors which impact retinal thickness measurements in children include birth weight, prematurity, maternal gestational hypertension, diabetes and smoking, and body mass index [21–25]. However, while these factors may explain some of the variation found in the current study, most have been shown to influence retinal thickness by less than < 6 μm [22–25] and are therefore unlikely to explain the larger difference found between Aboriginal and/or Torres Strait Islander children and non-Indigenous children in this study.

The thinner retina of Aboriginal and/or Torres Strait Islander children has a number of important implications. Unless this difference is considered when interpreting OCT data, abnormalities in retinal thickness may not be detected, hindering early intervention of sight-threatening ocular diseases or potentially triggering unnecessary referral. Perhaps more importantly, the reduced retinal thickness of Aboriginal and/or Torres Strait Islander children may influence their susceptibility to ocular disease later in life. It is possible that this structurally thinner retina, which potentially reflects reduced cell density in more than one layer, may have a reduced ability to withstand the insult of pathogenic factors such as elevated IOP or metabolic stress, however this association has not been explored and therefore remains speculative. These potential implications highlight the need to extend this research to Aboriginal and/or Torres Strait Islander adults, who are more likely to be affected by ocular diseases such as diabetic retinopathy [26], but whose retinal structure has not been characterized using high-resolution OCT imaging.

A small but statistically significant increase in TRT was observed in both the foveal and parafoveal zones with age in both groups, with the greatest change occurring in the foveal zone. This finding is consistent with the foveal region being the last macular zone to mature in childhood, as shown in histological studies [27]. Similarly, in a longitudinal study of non-Indigenous Australian children, Read, Alonso-Caneiro (2017) reported a 0.7 μm per year increase in foveal zone thickness over 18 months [19]. While this association reached statistical significance, the magnitude of change per year is very small and unlikely to be of clinical significance. Therefore, it can be concluded that retinal thickness, and the thickness of the individual retinal layers remained relatively stable within this age group. The finding that Aboriginal and/or Torres Strait Islander children and non-Indigenous children exhibited a similar rate of change in retinal thickness with age suggests that the differences in mean retinal thickness exhibited between groups were established from a young age and remained relatively consistent across the age range of children examined in this study.

In this group of primarily hyperopic children, there was no association between either axial length or SER and magnification corrected macula retinal thickness. It is likely that this lack of association was due to the small number of myopic children in the sample (n = 4), and hence relatively small range of axial lengths. In contrast, numerous previous studies have shown that a longer axial length and more myopic refractive error were associated with reduced parafoveal and perifoveal retinal zone thickness [4, 6, 19, 28].

This is the first study to report on retinal thickness measurements in Aboriginal and/or Torres Strait Islander peoples, however the findings should be interpreted in light of some limitations. Firstly, the topographic mapping of the retina was derived from six radial line scans and not mapped across the entire retina using a high-density raster volumetric imaging protocol. This scan type was used in this paediatric population due to its rapid acquisition time, in order to encourage more accurate and steadier fixation and hence improve image quality compared to a volumetric scan which involves a considerably longer acquisition time. Secondly, future studies are needed to assess potential systemic or environmental factors associated with retinal thickness (such as birth weight and body mass index) which were outside the scope of the current study.

In summary, the thinner macular retina exhibited by Australian Aboriginal and/or Torres Strait Islander children in this study is an important finding and should be considered when interpreting OCT findings in this population. Given the increasing prevalence of type 2 diabetes among Aboriginal and/or Torres Strait Islander children and adolescents, and the subsequent risk of diabetic retinopathy, the development of age and ethnicity-specific normative databases for retinal thickness will improve sensitivity for detecting small magnitude changes in retinal thickness that occur in the early stages of diseases such as diabetic retinopathy in this population. Future research is needed to not only investigate the factors which may contribute to this difference in retinal thickness, but also to determine whether such differences are associated with increased susceptibility to retinal disease later in life in this population.

## Supporting information

S1 File Inclusivity in global research. (DOCX)

S1 Data (XLSX)
